# Genetic analysis of the merozoite surface protein-1 block 2 allelic types in *Plasmodium falciparum *clinical isolates from Lao PDR

**DOI:** 10.1186/1475-2875-10-371

**Published:** 2011-12-17

**Authors:** Naly Khaminsou, Onanong Kritpetcharat, Jureerut Daduang, Lertchai Charerntanyarak, Panutas Kritpetcharat

**Affiliations:** 1 Faculty of Medical Technology, University of Health Science, Vientiane, Lao PDR; 2 Department of Pathology, Faculty of Medicine, Khon Kaen University, 123 Mittraphap Highway, Khon Kaen 40002, Thailand; 3 Department of Clinical Chemistry, Faculty of Associated Medical Sciences, Khon Kaen University, 123 Mittraphap Highway, Khon Kaen 40002, Thailand; 4 Department of Epidemiology, Faculty of Public Health, Khon Kaen University, 123 Mittraphap Highway, Khon Kaen 40002, Thailand; 5 Department of Clinical Microscopy, Faculty of Associated Medical Sciences, Khon Kaen University, 123 Mittraphap Highway, Khon Kaen 40002, Thailand

## Abstract

**Background:**

MSP-1 is one of the potential malarial vaccine candidate antigens. However, extensive genetic polymorphism of this antigen in the field isolates of *Plasmodium falciparum *represents a major hindrance for the development of an effective vaccine. Therefore, this study aimed to establish the prevalence and genetic polymorphisms of K1, MAD20 and RO33 allelic types of *msp-1 *block 2 among *P. falciparum *clinical isolates from Lao PDR.

**Methods:**

*Plasmodium falciparum *isolates were collected from 230 *P. falciparum*-infected blood samples from three regions of Lao PDR. K1, MAD20 and RO33 were detected by nested PCR; SSCP was used for polymorphism screening. The nested PCR products of each K1, MAD20 and RO33 allelic types that had different banding patterns by SSCP, were sequenced.

**Results:**

The overall prevalence of K1, MAD20 and RO33 allelic types in *P. falciparum *isolates from Lao PDR were 66.95%, 46.52% and 31.30%, respectively, of samples under study. Single infections with K1, MAD20 and RO33 allelic types were 27.83%, 11.74% and 5.22%, respectively; the remainders were multiple clonal infections. Neither parasite density nor age was related to MOI. Sequence analysis revealed that there were 11 different types of K1, eight different types of MAD20, and 7 different types of RO33. Most of them were regional specific, except type 1 of each allelic type was common found in 3 regions under study.

**Conclusions:**

Genetic polymorphism with diverse allele types was identified in *msp-1 *block 2 among *P*. *falciparum *clinical isolates in Lao PDR. A rather high level of multiple clonal infections was also observed but the multiplicity of infection was rather low as not exceed 2.0. This basic data are useful for treatment and malaria control program in Lao PDR.

## Background

Malaria remains one of the most important health-threatening parasitic diseases in tropical and subtropical areas, such as Lao PDR and other countries in Southeast Asia. In Lao PDR, many highly endemic areas exist, especially in the rural areas of Xekong, Attapeu, Savannakhet, Saravane, and Champasack provinces [1-3]. *Plasmodium falciparum *is responsible for most of the mortality, while *Plasmodium vivax *causes considerable morbidity [1,3,4]. Despite the enormous efforts that have been directed toward malaria control and prevention, multiple factors-including insecticide resistance in anopheline vectors, the lack of effective vaccines, and the emergence and rapid spread of drug-resistant strains-are the major problems for controlling and prevention of malaria. Therefore, the development of an effective malaria vaccine is urgently needed. However, extensive the genetic diversity in natural malaria parasite populations is a major obstacle for the development of an effective vaccine against these parasites, because antigenic diversity limits the efficiency of acquired protective immunity to malaria [5-7]. Many malarial proteins have been proposed for use as vaccine candidate antigens, but merozoite surface protein-1 (MSP-1) is the most used [8,9]. However, extensive genetic polymorphisms of the MSP-1 gene have been identified in *P*. *falciparum *isolates worldwide; this has caused extensive antigenic polymorphism [10,11]. It is important to investigate the diversity of *msp-1 *gene, in different geographic areas for the further development of effective malaria prevention and control.

MSP-1, with an approximate molecular size of 190 kDa, is a major surface protein of *P*. *falciparum*, and plays a role in erythrocyte invasion by the merozoite [12]. The protein is a principal target of human immune responses [13-15] and is a vaccine candidate antigen for blood stages [12-16]. The *msp-1 *gene has 7 variable blocks that are separated either by semi-conserved or conserved regions. Block 2, a region near the N-terminal of this gene, is the most polymorphic part of the protein and appears to be under the strongest diversifying selection within natural populations [12]. At present, four different allelic types of block 2 have been identified, including K1, MAD20, RO33 and MR [17,18].

The overall prevalence of malarial infection in Lao PDR recoded in 2008 by the Central of Malaria, Parasitology and Entomology of Lao PDR was 15% [Unpublished data]. At present, there is no data about the genetic diversity of *P. falciparum *in Lao PDR. For this reason, it is important to investigate the diversity of *msp-1 *gene, in different geographic areas for the further development of effective malaria prevention and control.

This study aimed to establish the prevalence and genetic polymorphisms of K1, MAD20 and RO33 allelic types of the *msp-1 *gene among *P. falciparum *field isolates from Lao PDR.

## Methods

### Study areas

**Figure 1:**
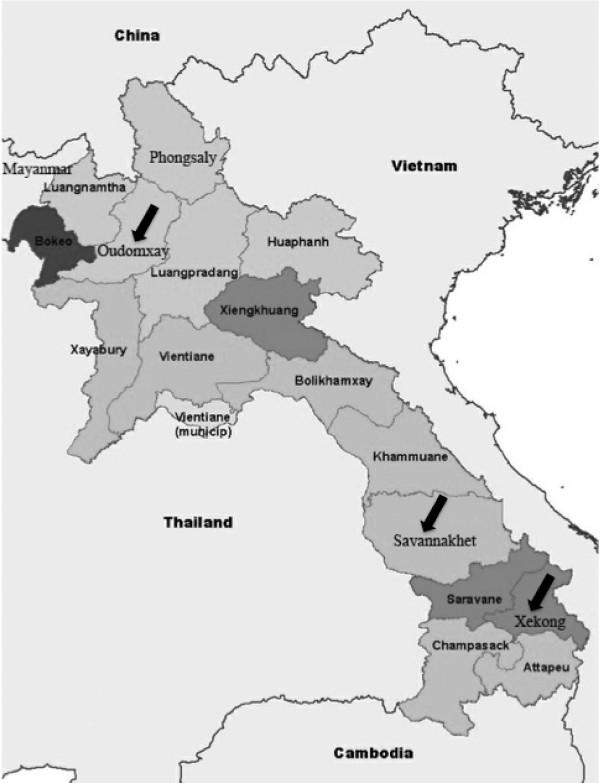
**Lao PDR map**. Arrows indicate blood sample collection sites: Oudomxay province represented the northern region, Savannakhet the central region, and Xekong the southern region.

Oudomxay province is situated in northern region; there is a 15-kilometer border with the autonomous area of Xishuangbanna of the People's Republic of China. Some 23 ethnic minorities mainly Hmong, Akha and Khamu populate Oudomxay. The prevalence of malarial infection in 2008 recorded by the Central of Malaria, Parasitology and Entomology of Lao PDR of this area was 0.9% [unpublished data]. Savannakhet province is situated on the banks of the Mekong River opposite Mukdahan province in Thailand. The province located in central region and bridges the country between Thailand and Vietnam. There are 11 ethnic minorities include Lowland Lao, Phoutai, Thai Dam, Katang, Mongkong, Vali, Lava, Soui, Kapo, Kaleung and Ta-Oi. The prevalence of malarial infection in Savannakhet was 9.4% in 2008. Xekong province is situated in southern region; has common border Vietnam to the east. Less than 5% of the population is ethnic Lao. The vast majority comes from one of at least 14 distinct ethnic minority groups. The main ethnic groups are Alak, Katu, Tarieng and Nge/Krieng. The prevalence of malarial infection in Xekong was 5% in 2008. The map of this study area was shown in Figure 1.

### Blood samples and genomic DNA extraction

*Plasmodium falciparum *isolates were collected from microscopically diagnosed *P. falciparum*-positive subjects (from thin and/or thick Giemsa-stained blood films) in three regions of Lao PDR: the northern region, in Oudomxay province; the central region, in Savannakhet province; and the southern region, in Xekong province (Figure 1) and demographics of this study was shown in Table 1. Blood was spotted on Whatman No. 3 filter paper strips by pricking a finger or using the leftover EDTA blood samples from routine work (after obtaining the consent of parents or guardians in the case of children). The 230 samples-15 from Oudomxay, 140 from Savannakhet and 75 from Xekong-were randomly collected during July 2008 to May 2009. The National Ethics Committee, Lao PDR, and the Institutional Ethics Board at Khon Kaen University, Thailand approved this study. All dried blood samples were extracted for genomic DNA using the Chelex-100 method, as described by Bereczky et al. [19]. The parasitaemia of blood was the number of parasites per μl of blood in a thick smear assuming a leukocyte count of 8,000 cells/μl [20].

**Table 1:** Demographics of the studied population

Characteristics	Oudomxay No. (%)	Savannakhet No. (%)	Xekong No. (%)	Total No. (%)
Gender				
Male	10 (4.35)	61 (26.52)	45 (19.57)	116 (50.43)
Female	5 (2.17)	79 (34.35)	30 (13.04)	114 (49.57)
Age group				
< 5	6 (2.61)	51 (22.17)	19 (8.26)	76 (33.04)
5-19	8 (3.48)	59 (25.65)	27 (11.74)	94 (40.87)
20-39	1 (0.43)	21 (9.13)	17 (7.39)	39 (16.96)
40-59	0	9 (3.91)	10 (4.35)	19 (8.26)
≥ 60	0	0	2 (0.87)	2 (0.87)

### Nested PCR for *Plasmodium falciparum*

All extracted DNA samples were confirmed for Plasmodium infection by using nested PCR, as described by Snounou et al. [21]. Genus-specific primers and species-specific primers of *P. falciparum*, *P. vivax*, *Plasmodium malariae *and *Plasmodium ovale *were used in this study. Blood samples infected by Plasmodium species other than *P. falciparum*, or by mixed species, were excluded.

### Nested PCR for *msp-1 *block 2, SSCP for polymorphism screening and DNA sequencing

The *msp-1 *block 2 allelic types (K1, MAD20 and RO33) of *P. falciparum *isolates were detected using the method described in Aubouy et al. [22]. The primers for *msp-1 *used in this study are shown in Table 2.

**Table 2:** Sequences of oligonucleotide primers used to amplify *msp-1 *block 2 (K1, MAD20 and RO33) allelic types of *Plasmodium falciparum *[22]

Primers	Sequences	Note
msp1-C1	5'-AAGCTTTAGAAGATGCAGTATTGAC-3'	Conserved
msp1-C2	5'-ATTCATTAATTTCTTCATATCCATC-3'	Conserved
K1a	5'-GAAATTACTACAAAAGGTGCAAGTG-3'	K1 family-specific
K1b	5'-AGATGAAGTATTTGAACGAGGTAAAGTG-3'	K1 family-specific
MAD20a	5'-AAATGAAGGAACAAGTGGAACAGCTGTTAC-3'	MAD20 family-specific
MAD20b	5'-ATCTGAAGGATTTGTACGTCTTGAATTACC-3'	MAD20 family-specific
RO33a	5'-TAAAGGATGGAGCAAATACTCAAGTTGTTG-3'	RO33 family-specific
RO33b	5'-CATCTGAAGGATTTGCAGCACCTGGAGATC-3'	RO33 family-specific

The first round of PCR was done with the external 5' and 3' primers, which amplified *msp-1 *block 2 plus some of the flanking regions. The first round of amplification was carried out in 25 μl of reaction mix containing: 1× PCR, 4.0 mM MgCl_2_, 0.1 mg/ml gelatin, 0.05% Triton X-100, 200 μM dNTP mix, 0.8 U Taq polymerase, 75 nM of each conserved primer, distilled water up to 20 μl, and 5 μl of DNA template. Amplification was performed using the following conditions: 94°C for 5 min, 35 cycles at 94°C for 30 s, 58°C for 1 min, and 72°C for 1 min, followed by extension at 72°C for 10 min.

The second round of PCR was performed in three separate tubes each containing a single pair of K1, MAD20 and RO33 allele-specific primers. The 25 μl reaction mix contained 1× PCR, 4.0 mM MgCl_2_, 0.1 mg/ml gelatin, 0.05% Triton X-100, 200 μM dNTP mix, 0.8 U Taq polymerase, 75 nM of each allele-specific primer pair (K1a/K1b, MAD20a/MAD20b or RO33a/RO33b), distilled water up to 20 μl, and 5 μl of the first-round PCR product. Amplification cycles for second round PCR were initial denaturation for 5 min at 94°C, followed by 35 cycles of 30 s denaturation at 94°C, 1 min annealing at 58°C, and extension at 72°C for 1 min. Final extension was carried out at 72°C for 10 min. PCR products were visualized by UV transillumination at 302 nm on gel documentation system after electrophoresis on 2% agarose gel (Promega/Boe-hringer) using 0.5 × TBE buffer at 100 volts.

Each DNA product was screened for polymorphic banding using SSCP, as described in Huby-Chilton et al. [23], in duplicate: one stained with silver nitrate and the other with ethidium bromide. The K1, MAD20 and RO33 products from nested PCR that showed normal and polymorphic bands were sent for DNA sequencing by using each forward and reverse primer pair.

### Statistical and DNA sequence analysis

A two-proportions test was used to compare the prevalence of *msp-1 *block 2 allelic types between age groups, between genders and between parasitaemic groups. To understand the identity of *msp-1 *block 2 allelic types of Lao PDR with respect to isolates of other regions worldwide, sequence data available in the public domain were downloaded for the following *msp-1 *allelic types: 3D7 (K1-XM 001352134); Thailand (K1-AB276018, K1-AB276008, MAD20-AB276013, MAD20-AB276006, RO33-AB276015, RO33-M77737); Vietnam (K1-AF509651, MAD20-AF509699, MAD20-AF509696, MAD20-AF509694); Cambodia (K1-HM153247, MAD20-HM153249); Indonesia (K1-AF191061, RO33-AF191064); India (K1-DQ485421, K1-DQ485424, RO33-JF300128); Myanmar (K1-GQ861445, K1-EU445566, MAD20-EU445560, MAD20-EU445555); Ghana (RO33-AB276005); Malawi (K1-HM153200, MAD20-HM153237, RO33-HM153239, RO33-HM153223); Gambia (MAD20-AB276004); Peru (K1-FJ612037, K1-FJ612017, RO33-FJ612064); Tanzania (MAD20-AF061147, RO33-AF061150); Brazil (MAD20-AF509667, RO33-AB276002); Iran (RO33-AY138508); Sudan (RO33-AB300615); and western Africa (RO33-M55001). DNA sequence data for each allelic family was aligned together with other allelic sequences worldwide by using the ClustalW program through BioEdit 5.0.7 software; phylogenetic trees were created using MEGA 5 [24].

## Results

The distribution of *msp-1 *block 2 allelic types in *P. falciparum *field isolates from Lao PDR was determined, based on microscopic diagnosis, from a total of 230 blood samples randomly collected from *P. falciparum*-infected patients who attended regional malarial clinics in three geographic areas of Lao PDR from July 2008 to May 2009: in Oudomxay province (representing the northern region), Savannakhet province (central), and Xekong province (southern). The patients consisted of 116 (50.43%) males and 114 (49.57%) females: 76 (33.04%) children younger than 5 years of age; 94 (40.87%) patients whose ages ranged 5-19 years; 39 (16.96%) patients whose ages ranged 20-39 years; 19 (8.26%) patients whose ages ranged 40-59 years; and 2 (0.87%) patients whose ages ≥ 60 years. These blood samples were confirmed for *P. falciparum *infection.

All blood samples were typed for *msp-1 *block 2 K1, MAD20 and RO33 allelic types by nested PCR. The results showed that 208 (90.43%) blood samples were positive for *msp-1 *block 2 allelic types, while 22 (9.57%) were negative. Of the total blood samples, 64 (27.83%) were positive for only the K1 allelic type, 27 (11.74%) for MAD20 only, and 12 (5.22%) for RO33 only (Table 3). The proportion of multiclonal isolates (multiple clonal infections) and MOI in different study areas is also given in Table 3. The total of multiple clonal infections in the three regions of Lao PDR was 45.65% (105/230 samples), and the proportion of multiclonal isolates ranged from 26.67% (20/75) in Xekong (southern region) to 55.71% (78/140) in Savannakhet (central region). Of the 105 samples showing mixed-allelic types of infection: 45 (19.57% of overall samples) were K1 + MAD20; 25 (10.87%) K1 + RO33; 15 (6.52%) MAD20 + RO33, and 20 (8.70%) K1 + MAD20 + RO33 (Table 3). The prevalence of total K1, MAD20 and RO33 allelic types was 66.96% (154/230), 46.52% (107/230) and 31.30% (72/230), respectively, of the study samples. Complexity of infection (multiplicity of infection, MOI) was estimated by dividing the total number of fragments detected in the individual system by the number of samples positive in this study. MOI was highest (1.7) in Savannakhet province and lowest in Oudomxay province (1.2). The estimated MOI of all studied area was 1.6. Clonal fluctuation in each K1, MAD20 and RO33 allelic type was not observed in *P. falciparum *clinical isolates under study, so that all the banding patterns on nested PCR of each allelic type was monomorphic bands as about 200 pb for K1, 180 bp for MAD20 and 150 for RO33.

**Table 3:** Prevalence of *msp-1 *block 2 allelic types in Lao PDR

*msp-1 *block 2 allelic type	Oudomxay No. (%)	Savannakhet No. (%)	Xekong No. (%)	Total No. (%)
K1	1(6.66)	39(27.86)	24(32.00)	64(27.83)
MAD20	4(26.66)	9(6.43)	14(18.67)	27(11.74)
RO33	1(6.66)	8(5.71)	3(4.00)	12(5.22)
K1 + MAD20	2(13.34)	35(25.00)	8(10.66)	45(19.57)
K1 + RO33	2(13.34)	18(12.86)	5(6.66)	25(10.87)
MAD20 + RO33	2(13.34)	8(5.71)	5(6.66)	15(6.52)
K1 + MAD20 + RO33	1(6.66)	17(12.14)	2(2.66)	20(8.70)
Negative	2(13.34)	6(4.29)	14(18.67)	22(9.57)

Total	15(100.00)	140(100.00)	75(100.00)	230(100.00)
Total K1	6 (40.00)	109(77.86)	39(52.00)	154(66.96)
Total MAD20	9(60.00)	69(49.29)	29(38.67)	107(46.52)
Total RO33	6(40.00)	51(36.43)	15(20.00)	72(31.30)
Multiclonal isolates	7 (46.67)	78 (55.71)	20(26.67)	105(45.65)
MOI	1.2	1.7	1.4	1.6

MOI-Multiplicity of Infection (Number of clones identified as *msp1 *allelic types, excluding PCR negatives)

Age group distribution of *P. falciparum*-infected patients under study, and the distribution of *msp-1 *block 2 allelic types of *P. falciparum *field isolates among age groups, is shown in Table 4. When observing the distribution of these allelic types in term of two age groupings-<5 years and ≥ 5 years-it was found that the prevalence of K1, MAD20 and RO33 allelic types in < 5 years age group and in ≥ 5 years age group was not significantly different (*p > 0.05*, two-proportions test). The prevalence of multiple clonal infections with K1 and MAD20 allelic types in < 5 years age group was significantly higher than in ≥ 5 years age group (*p = 0.017*, two-proportions test); while the prevalence of multiple clonal infections with MAD20 and RO33 allelic types was significantly higher in the ≥ 5 years age group than in the < 5 years age group (*p = 0.043*, two-proportions test). However, the prevalence of total K1, total MAD20 and total RO33 in the two age groups was not significantly different (*p *> 0.05, two-proportions test). MOI was not different among age groups, but the highest MOI (1.8) was found in 40-95 years age group. The prevalence of K1, MAD20, RO33 and multiple clonal infections, and the overall total of each allelic type in male and female, was not significantly different (*p > 0.05*, two-proportions test).

**Table 4:** Distribution of K1, MAD20 and RO33 allelic types among age groups

	Age group (years)					
		
*msp-1 *block 2 allelic type	< 5 No. (%)	5-19 No. (%)	20-39 No. (%)	40-59 No. (%)	≥ 60 No. (%)	Total No. (%)
K1	16 (7.69)	29 (13.94)	13 (6.25)	5 (2.40)	1 (0.48)	64 (30.77)
MAD20	9 (4.33)	12 (5.77)	4 (1.92)	2 (0.96)	0	27 (12.98)
RO33	5 (2.40)	6 (2.88)	1 (0.48)	0	0	12 (5.77)
K1 + MAD20	22 (10.58)	15 (7.21)	4 (1.92)	4 (1.92)	0	45 (21.63)
K1 + RO33	8 (3.85)	12 (5.77)	4 (1.92)	1 (0.48)	0	25 (12.02)
MAD20 + RO33	2 (0.96)	6 (2.88)	5 (2.40)	2 (0.96)	0	15 (7.21)
K1 + MAD20 + RO33	7 (3.37)	7 (3.37)	3 (1.44)	3 (1.44)	0	20 (9.62)
Multiclonal isolates	39 (18.75)	40 (19.23)	16 (7.69)	10 (4.81)	1 (0.48)	106 (50.96)
MOI	1.7	1.5	1.6	1.8	1.0	1.6

Total	69 (33.17)	87 (41.83)	34 (16.35)	17 (8.17)	1 (0.48)	208 (100.00)

The distribution of K1, MAD20 and RO33 by estimated parasitaemia is shown in Table 5. This study found that the distribution of K1, MAD20, RO33 and multiple clonal infections was not significantly different between < 480 parasites/μl and ≥ 480 parasites/μl (*p > 0.05*, two-proportion test). Multiplicity of *P. falciparum *infection was not correlated with the estimated parasitaemia.

**Table 5:** Distribution of *msp-1 *block 2 allelic types among parasitaemic groups from 3 regions of Lao PDR

*msp-1 *block 2 allelic type	Parasitaemia (cells/μl) Number of cases (%)				Total
		
	≤ 80	120-480	520-960	≥ 1000	
K1	6 (2.88)	18 (8.65)	30 (14.42)	10 (4.80)	64 (30.76)
MAD20	1 (0.48)	7 (3.36)	14 (6.73)	5 (2.40)	27 (12.98)
RO33	2 (0.96)	4 (1.92)	6 (2.88)	0	12 (5.76)
K1 + MAD20	3 (1.44)	12 (5.76)	19 (9.13)	11 (5.28)	45 (21.63)
K1 + RO33	3 (1.44)	6 (2.88)	12 (5.76)	4 (1.92)	25 (12.01)
MAD20 + RO33	0	5 (2.40)	5 (2.40)	5 (2.40)	15 (7.21)
K1 + MAD20 + RO33	2 (0.96)	9 (4.32)	8 (3.84)	1 (0.48)	20 (9.61)
Multiclonal isolates	8 (3.84)	32 (15.38)	44 (21.15)	21 (10.09)	105 (50.48)

MOI	1.6	1.7	1.6	1.6	1.6

Total	17 (8.17)	61 (29.32)	94 (45.19)	36 (17.30)	208 (100.00)

The nested PCR product of each K1, MAD20 and RO33 allelic type was screened for polymorphism by SSCP. The results showed that the total polymorphic alleles (mutations) of K1, MAD20 and RO33 found in *P. falciparum *isolates from Lao PDR were: 19/154 (12.34%) of total K1 alleles found in *P. falciparum *field isolates, 12/107 (11.21%) of total MAD20 alleles, and 6/72 (8.33%) of total RO33 alleles, as shown in Table 6. In Oudomxay, the polymorphic alleles of K1 comprised 16.67% (1/6) of total K1 alleles, and 11.11% (1/9) of total MAD20; but no polymorphic alleles of RO33 were found. In Savannakhet, the polymorphic alleles of K1, MAD20 and RO33 found in *P. falciparum *were 11.93% (13/109) of total K1 alleles, 11.56% (8/69) of total MAD20, and 7.84% (4/51) of total RO33. Finally, the polymorphic alleles of K1, MAD20, and RO33 found in *P. falciparum *isolates from Xekong were 12.82% (5/39) of total K1 alleles, 10.34% (3/29) of total MAD20, and 13.33% (2/15) of total RO33.

**Table 6:** The polymorphic alleles of K1, MAD20, and RO33 results by SSCP screening of *P. falciparum *field isolates from Oudomxay, Savannakhet, and Xekong

*msp-1 *block 2 allelic type	Oudomxay	Savannakhet	Xekong	Total
	
	% (polymorphic alleles/total)	% (polymorphic alleles/total)	% (polymorphic alleles/total)	% (polymorphic alleles/total)
K1	16.67 (1/6)	11.93 (13/109)	12.82 (5/39)	12.34 (19/154)
MAD20	11.11 (1/9)	11.59 (8/69)	10.34 (3/29)	11.21 (12/107)
RO33	0 (0/6)	7.84 (4/51)	13.33 (2/15)	8.33 (6/72)
Total polymorphic alleles	13.33 (2/15)	10.92 (25/229)	12.05 (10/83)	11.11 (37/333)

**Figure 2:**
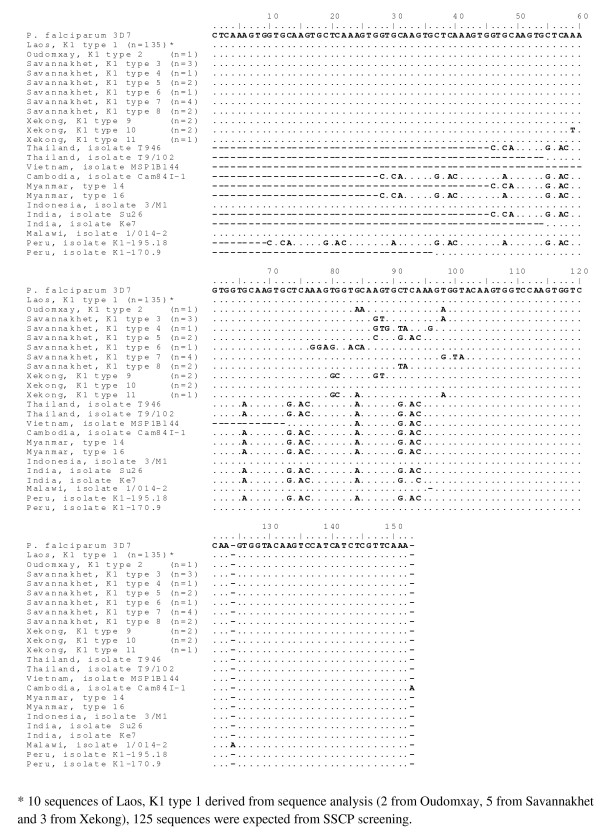
**DNA sequences of *msp-1 *block 2 K1 allelic types from Lao PDR aligned with K1 allelic type of *P. falciparum *3D7**.

A total of 11 different allelic types of K1 were recognized by sequence alignment with *P*. *falciparum *3D7 (XM 001352134) as shown in Figure 2. "Laos, K1 type 1" is common found in 3 regions under study, this normal sequence is 100% identity to *P. falciparum *3D7 (XM 001352134). The remainders were regional specific, named as "Oudomxay, K1 type 2"; "Savannakhet, K1 type 3-8" and "Xekong K1 type 9-11".

**Figure 3:**
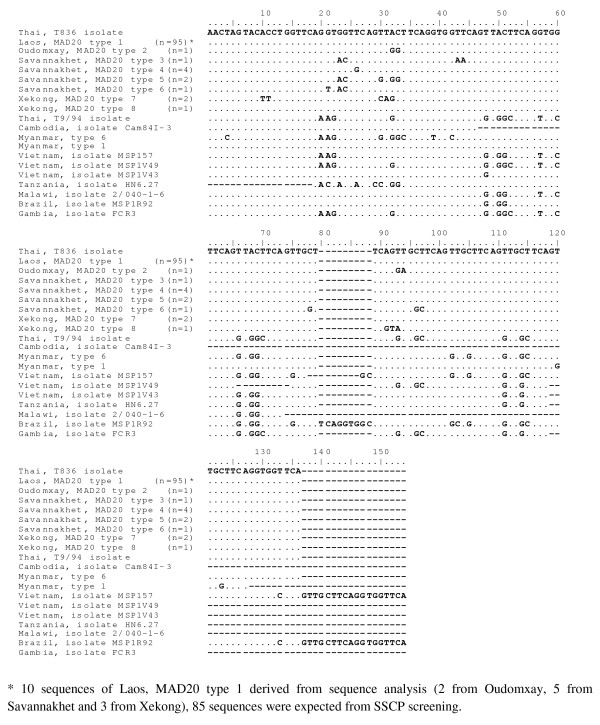
**DNA sequences of *msp-1 *block 2 MAD20 allelic types from Lao PDR aligned with MAD20 allelic type of *P. falciparum *Thai, T836 isolate**.

A total of eight different alleles of MAD20 were recognized by sequence alignment with *P*. *falciparum *T836 isolate (AB276013). This MAD20 sequences from Lao PDR aligned together with other MAD20 sequences from other regions worldwide, is shown in Figure 3. "Laos, MAD20 type 1" is common normal sequence 100% identity to *P. falciparum *T836 isolate (AB276013). The remainders were regional specific, named as "Oudomxay, MAD20 type 2", "Savannakhet, MAD20 type 3-6" and "Xekong, MAD20 type 7-8".

**Figure 4:**
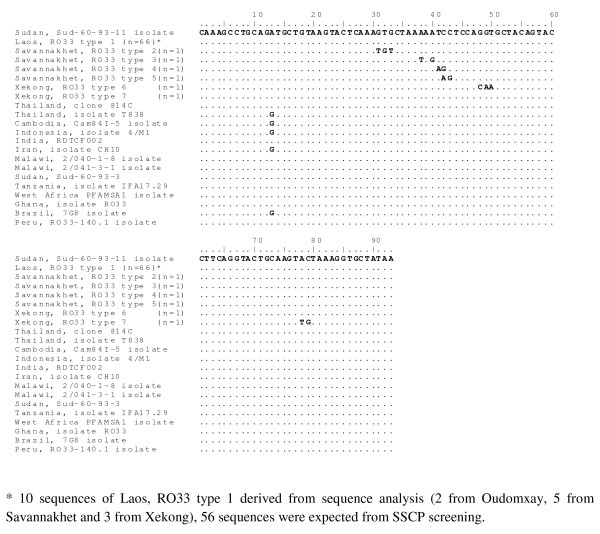
**DNA sequences of *msp-1 *block 2 RO33 allelic types from Lao PDR aligned with RO33 allelic type of *P. falciparum *Thai, Sudan, Sud-60-93-11 isolate**.

Sequence analysis for RO33 allelic types from Lao PDR revealed that there were 7 different alleles recognized by alignment with *P. falciparum *Sud-60-93-11 isolate (AB300615). "Lao, RO33 type 1" is common normal sequence 100% identity to *P. falciparum *Sud-60-93-11 isolate (AB300615). The remainders were also regional specific, named as "Savannakhet, RO33 type 2-5" and "Xekong, RO33 type 6-7". This RO33 sequences aligned together with RO33 from other regions is shown in Figure 4.

**Figure 5:**
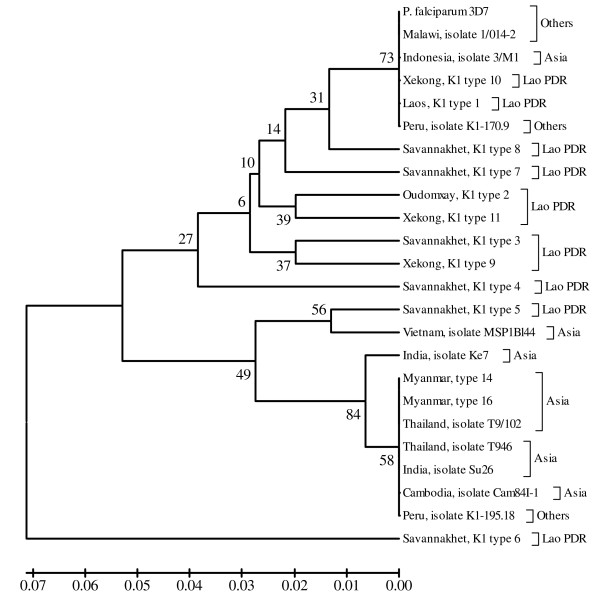
**Phylogenetic tree of described *P. falciparum *field isolates from Lao PDR and other regions worldwide based on their K1 allelic type**. The evolutionary history was inferred using the UPGMA method. The optimal tree with the sum of branch length = 0.40987732 is shown. The percentage of replicate trees in which the taxa clustered together in the bootstrap test (1,000 replicates) is shown.

**Figure 6:**
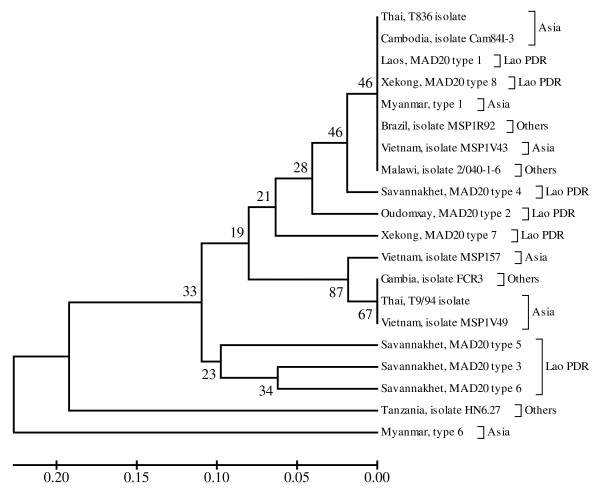
**Phylogenetic tree of described *P. falciparum *field isolates from Lao PDR and other regions worldwide based on their MAD20 allelic type sequences**. The evolutionary history was inferred using the UPGMA method. The optimal tree with the sum of branch length was 1.13478897. The percentage of replicate trees in which the taxa clustered together in the bootstrap test (1,000 replicates) is shown.

**Figure 7:**
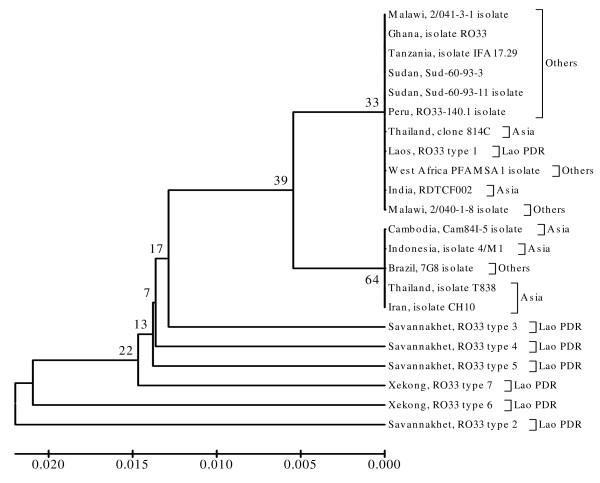
**Phylogenetic tree of described *P. falciparum *field isolates from Lao PDR and other regions worldwide based on RO33 allelic type**. The evolutionary history was inferred using the UPGMA method. The optimal tree with the sum of branch length was 0.12513671. The percentage of replicate trees in which the taxa clustered together in the bootstrap test (1,000 replicates) is shown.

Finally, each phylogenetic tree of K1, MAD20 and RO33 sequences from Lao PDR, constructed together with K1, MAD20 and RO33 sequences from other regions worldwide, showed that some K1 allelic type sequences from Lao PDR were grouped together with K1 allelic type sequences from other regions. The K1 allelic type sequence from Lao PDR known as Savannakhet isolate type 6 was unique, and could not be grouped together with other K1 allelic type sequences from Lao PDR or other regions (Figure 5). The phylogenetic tree of MAD20 allelic type sequences from Lao PDR and from other regions showed that they were grouped together with the monophyletic clade in only 34% of bootstrap replications (Figure 6). Most of RO33 allelic type sequences from Lao PDR, however (except Laos, RO33 type 1), were not grouped together with RO33 allelic type sequences from other regions worldwide (Figure 7).

## Discussion

Human malaria is an infectious disease transmitted by the bite of mosquitoes infected with any of five Plasmodium species: *P. falciparum*, *P. vivax*, *P. malariae*, *P. ovale *and *Plasmodium knowlesi*. Among these species, *P. falciparum *can cause various clinical problems including heart, lung, kidney and brain damage, and possibly death. *P. falciparum *remains an important public health concern because of its increasing resistance to common anti-malarial drugs, resulting in a high mortality rate. In malaria-endemic areas, infections by multiple parasite clones are frequent, and clone fluctuation may be a logical strategy for the appearance of strain-specific responses [25-28].

This study investigated the prevalence and polymorphism of *P. falciparum *subpopulations in three regions of Lao PDR by nested PCR. This nested PCR method cannot be used to distinguish alleles nearly identical or identical in size, but differing in sequences [29]. Polymorphic banding by size of all parasite isolates in this study was not observed by agarose gel electrophoresis of the nested PCR products because all isolates were the same or very nearly the same size. To distinguish polymorphism in allele-specific families in this study, all DNA products from each allele-specific family detected by nested PCR were screened by SSCP. The different band patterns from SSCP were selected, and these nested PCR products were sequenced. This study found that sequences that had only one nucleotide base change could produce a different banding pattern on SSCP, as seen in Xekong isolate K1 type 10 and Savannakhet isolate MAD20 type 4. Moreover, nested PCR could not detect *P. falciparum *isolates, which have polymorphic allele sequences because of insertion or deletion of a few nucleotide bases. The prevalence of polymorphic strains found in each *msp-1 *block 2 allelic type in this study may be underestimated because of variations in sensitivity which occurred throughout the experiment due to weak bands, as well as in the estimation of band length, as suggested by Aubouy et al. [22].

Nevertheless, the *P. falciparum *isolate K1 allelic type was found to be the most prevalent in Lao PDR, as is the case in other regions worldwide: K1, MAD20 and RO33 allelic types comprised 66.95%, 46.52% and 31.30%, respectively, of samples in the present study. When observing the three allelic types in the three regions of Lao PDR separately, this study found that the MAD20 allelic type was more prevalent than the others in Oudomxay, the least malaria-endemic area of Lao PDR. However, the number of samples collected from Oudomxay was low due to the sampling formula, which depended on the prevalence of malaria infection in this region. The distribution of K1, MAD20 and RO33 allelic types in highly endemic areas such as Savannakhet and Xekong provinces had the same patterns and the same overall distribution as the total three regions.

Monomorphic banding of K1 and MA20 allelic types in Lao PDR observed on agarose gel electrophoresis may be due to limited samples group with clinical symptoms in this study, or due to the fact that clonal fluctuations in each *msp-1 *block 2 allelic types of *P. falciparum *isolate in Lao PDR is quite low. Moreover, it may be due to low parasite density in the blood samples under study (majority of parasite density < 1,000 cells/μl). Limited clonal fluctuation of K1 allelic type was also seen in urban malaria infected patients (200 and 220 bp) in Burkina Faso [30] and in Darjeeling district, West Bengal state of India (140 bp) [11]. Moreover, monomorphic band of MAD20 (220 bp) and RO33 (150/160 bp) were also found in some districts in eastern and northeastern India [11].

However, the total *P. falciparum msp-1 *block 2 genotypic isolates, multiple clonal infections and MOI in this study might be underestimated, because the new MR allele type, as described by Takala et al. [17], was not investigated. Takala et al. reported that the MR allele type in *P*. *falciparum *isolates in Asembo Bay, Kenya, was found in 29% of the samples. Happi et al. [18] found a proportion of K1 (79%), MAD20 (32%) and RO33 (38%) alleles in *P*. *falciparum *isolates from Nigeria. The frequency of MAD20 in the study by Happi et al. was lower than in this study and in studies from other regions. Aubouy et al. [22] studied the polymorphism in two MSP-1 and MSP-2 genes from Gabon. They found that MSP-1 alleles of K1, MAD20 and RO33 comprised 90.4%, 63.5% and 36.5% of the samples studied; they also found that *P. falciparum *polymorphism was extensive in southeast Gabon, and that most infections were composed of multiple clones. About 45.65% (105/230) of *P. falciparum *infections in Lao PDR in this study were multiple clonal infections of the three allele types studied. Kang et al. [10] studied genetic polymorphism of *msp-1 *and *msp-2 *in *P. falciparum *field isolates from Myanmar, and found that the K1 allele infection rate was 73.02% (46/63); but the majority (63.5%) occurred as multiple clonal infections with the MAD20 allele type. This study found that total multiple clonal infections of K1 and MAD20 (including K1 + MD20 + RO33) was lower than that found in Myanmar (28.26% vs. 63.5%), but that single infection with K1 was higher (27.8% vs. 9.5%). Otherwise, single infection with MAD20 in Lao PDR was lower than in Myanmar (11.7% vs. 27%).

MOI has been suggested to differ in relation to age, transmission intensity and parasite density [31-33]. MOI in Lao PDR was rather low as not exceed 2.0, and was not correlated with age group like the previous report by Vafa et al. [33]. The overall MOI in Lao PDR (1.6) was not different from the MOI reported in Vietnam (1.76) and Brazil (1.42), but lower than in Tanzania (2.37) and in Gabon (4.0) [22,34]. There were many reports showed that the MOI was correlated with parasite density. MOI in this study did not increase with higher parasite density, similar to those reports in Brazzaville, Republic of Congo and in Gabon [22,35]. This study used clinical *P. falciparum *isolates from patients with clinical symptoms, so that the MOI may be lower than that reported in asymptomatic children [36]. Many reported showed that MOI would reflect that infected individual has immunity against malaria [32,37]. Thus, the MOI should be studied in asymptomatic *P. falciparum*-infected population in Lao PDR further, especial in children.

The frequency of polymorphic alleles in the K1 allele type in Lao PDR and in Myanmar was not markedly different (12.34% vs. 9.5%), but the frequency of polymorphism in the MAD20 allelic type from Myanmar was much higher than that from Lao PDR (27% vs. 11.21%).

Lao PDR is a landlocked country with a long-space area. Ethnic minority populations are very localized distribution. Migration of the population within the country and cross-country is relatively low. Thus, the spread of polymorphic clones of *msp-1 *block2 allelic types of *P*. *falciparum *is limited to residents. Polymorphism of the K1, MAD20 and RO33 allelic types may occur in confined areas. Based on sequence data analysis and phylogenetic tree of each K1, MAD20 and RO33 allelic type sequences in Lao PDR with allelic type sequence data reported in other areas worldwide, found that Savannakhet, K1 type 6 and Savannakhet, RO33 type 2 may be unique polymorphic allelic types of *P. falciparum*-clinical isolate in Lao PDR.

## Conclusion

Genetic polymorphism with diverse allelic types was identified in *msp*-1 block 2 in *P*. *falciparum *clinical isolates from Lao PDR. A rather high level of multiple clonal infections was also observed, but the degree of multiplicity of infection was rather low as not exceed 2.0. Clonal fluctuation in each allelic type was not observed. Most of the polymorphic sequences of each K1, MAD20 and RO33 allelic type were regional specific. This basic genetic data of *msp-1 *block 2 allelic types is useful for treatment and malaria control program in Lao PDR.

## Competing interests

The authors declare that they have no competing interests.

## Authors' contributions

The work presented here was carried out in collaboration between all authors. NK, PK and OK were the principal investigators responsible for the study design, data collection and analysis, and manuscript preparation. PK and JD provided valuable technical guidance, and LC provided valuable statistical guidance. All authors read and approved the final version of the manuscript.
